# A Bioprocess Engineering Approach to Boost Selection of Fully Segregated Transformants in Cyanobacteria

**DOI:** 10.1002/bit.70024

**Published:** 2025-07-14

**Authors:** Cecilia Salvagnini, Eliana Gasparotto, Veronica Lucato, Elisabetta Bergantino, Matteo Ballottari, Elena Barbera, Nico Betterle, Eleonora Sforza

**Affiliations:** ^1^ Department of Industrial Engineering University of Padova Padua Italy; ^2^ Department of Biotechnology University of Verona Verona Italy; ^3^ Department of Biology University of Padova Padova Italy

**Keywords:** CSTR, cyanobacteria, homoplasmy, natural transformation, polyploidy, transformant selection

## Abstract

Cyanobacteria are photoautotrophic microorganisms with significant applications in biotechnology. Although many cyanobacteria, including *Picosynechococcus* sp. (formerly called *Synechococcus* sp.) PCC 11901 (*Picosynechococcus*) and *Synechocystis* sp. PCC 6803 (*Synechocystis*), are readily and naturally transformable, their polyploidy poses a major challenge. To obtain a stable phenotype, transgenic strains must be fully segregated, i.e. mutations must appear in all chromosome copies. Traditional protocols rely on re‐streaking of colonies on increasingly selective plates, a time‐intensive laboratory procedure that requires continuous intervention from the operator. This study proposes an alternative protocol that combines transformation in a batch system in liquid culture with transformant selection in a continuous‐flow stirred‐tank reactor system. This protocol led to the successful selection of homoplasmic transformants of *Picosynechococcus* containing, alternatively, an antibiotic resistance alone (construct “SmR”) or a more complex construct (“bKT”) that leads to the accumulation of a ketocarotenoid. The stability of SmR transformants under semi‐continuous cultivation in the absence of antibioticsf was tested for 42 days, proving their potential fitness to industrial cultivation conditions. The selection process was also validated on the model species *Synechocystis*, demonstrating its applicability to other cyanobacterial strains.

## 1 Introduction

Cyanobacteria are unique among prokaryotes for their ability to perform oxygenic photosynthesis. These microorganisms are notable for their potential in biotechnological applications, contributing to the sustainable production of bioactive compounds, such as food additives, biofuels, vitamins, enzymes, and pharmaceuticals (Abed et al. 2009). Due to their industrial potential, the transformability of cyanobacteria has been extensively investigated (Vioque 2007), however, researchers encounter a major drawback: polyploidy. Several species of cyanobacteria possess, in fact, multiple copies of their chromosomes (Watanabe 2020). This trait is believed to confer evolutionary advantages such as, among others, protection against mutation of essential genes and chromosomal damage from oxidative stress due to photosynthesis (Latifi et al. 2009). Among cyanobacteria, *Picosynechococcus* has been recently isolated from the Johor River estuary (Singapore) and appears to be a promising strain for industrial cultivation. This is due to its tolerance to wide ranges of temperature, salinity and light intensities, as well as to high biomass productivity. Although its genome has been sequenced, the exact number of chromosome copies has not yet been reported. The number of chromosome copies in cyanobacteria is not fixed, it can depend on growth phase (lag, exponential, linear) (Watanabe 2020) as well as availability of nutrients, particularly phosphorus (Pope et al. 2020). The effect of growth and nutrient conditions on the polyploidy of *Picosynechococcus* remains unexplored; however, recent studies suggest that it is a highly polyploid strain, as evidenced by the need of multiple re‐streaks on plates with high antibiotic concentrations (Mills et al. 2022). Polyploidy can complicate transformant generation, as mutations must occur in all copies of the chromosomes for stably maintaining transgene expression and resulting mutant phenotype (Pope et al. 2020). The most efficient genetic engineering tools and methods on *Picosynechococcus* are being extensively investigated; among methods, natural transformation seems the easiest way to obtain mutants, entailing the replacement of a non‐essential gene locus with the construct of interest or the direct insertion of the latter in a neutral genomic site. The gene of interest is typically delivered together with an antibiotic‐resistance cassette to select transformants, which initially contain both wild‐type and mutated chromosomes, a condition called “heteroplasmy” (Zekonyte et al. 2020). To obtain “homoplasmic” or “fully segregated” transformants, where all chromosome copies contain the transgene(s), colonies must be re‐streaked on media with increasing antibiotic concentrations, which is a time‐intensive procedure, requiring constant intervention from an operator manually picking the positive colonies. This procedure generally takes up to several weeks, depending on the cyanobacterial strain, mutation type and site of insertion (Lea‐Smith et al. 2016).

In this study, an alternative protocol for the transformation and selection of fully segregated transformants in lab‐scale (continuous stirred tank reactor [CSTR], also called as a chemostat) systems is proposed. The idea behind the use of the chemostat technology is that selection of transformants requires a selective environment, intrinsically provided by this reactor system (Gresham and Dunham 2014), which imposes specific growth rates to cells to remain inside the reactor. To obtain an extremely selective environment, two factors were combined: the increase of antibiotic concentration in the inlet medium, as it happens in serial re‐streaking, and the decrease in residence time, which imposes faster cellular growth rates. This allows only for the fittest population, hence the one containing the higher number of copies of the construct of interest, to endure inside the reactor, with the rest being washed out of it. This protocol was first tested on *Picosynechococcus* by introducing a construct containing only a spectinomycin‐resistance cassette (*smr*). A second trial involved the insertion of a more complex construct carrying a β‐carotene ketolase gene (*bkt*) alongside a kanamycin‐resistance cassette (*kmr*). Both trials successfully resulted in the selection of fully segregated transformants, hereafter referred to as SmR and bKT,‐ depending on the inserted recombinant DNA sequence, within approximately 2 weeks. Furthermore, the stability of SmR transformants was evaluated in a longterm semi‐continuous cultivation system in the absence of selective pressure given by antibiotics. Finally, the method was validated using the model species *Synechocystis*, known to contain several dozen copies of chromosomes, depending on growth phase, medium composition and light intensity (Zerulla et al. 2016), demonstrating the applicability of the novel method across different cyanobacterial species.

## 2 Materials and Methods

### 2.1 Cyanobacterial Strains and Growth

*Picosynechococcus* was kindly provided by Professor Peter J. Nixon (Imperial College London, UK). Cultures were grown in a modified version of MAD liquid medium (Włodarczyk et al. 2020) named “MAD_low P_” containing 29 mM NaNO_3_, 0.16 mM KH_2_PO_4_ and 55 µM FeCl_3_. Additionally, NaHCO_3_ 12 mM was used as buffer instead of Tris‐HCl to maintain a pH range of 7.5‐8. Solid medium was obtained by adding 1.2% (w/v) Agar and sodium thiosulfate 12.6 mM, as previously described (Włodarczyk et al. 2020).

*Synechocystis* was purchased from PCC (Pasteur Culture collection of Cyanobacteria, France) and cultured in modified BG11 liquid medium (Rippka et al. 1979), named “BG11_low P_” where HEPES was substituted with NaHCO_3_ 18 mM to maintain a pH range of 7.5‐8, and K_2_HPO_4_ was reduced to 0.18 mM. Petri dishes were obtained by adding 1.5% w/v Agar.

All culture flasks and agar plates were maintained at 30°C under continuous white LED light at 50 µmol photons/m²/s.

### 2.2 Generation of Recombinant Constructs in *Picosynechococcus*

The codon‐optimized nucleotide sequence encoding the β‐carotene 4‐ketolase (*bKT*) of *Chlamydomonas reinhardtii* (AY860820.1) was taken from previous work (Betterle et al. 2025; Perozeni et al. 2020; Włodarczyk et al. 2020). Selection cassettes conferring resistance to spectinomycin (*smr*) and kanamycin (*kmr*) were amplified respectively from plasmids pSW039 and pSW071 (Włodarczyk et al. 2020), and plasmids were obtained from the Addgene repository (https://www.addgene.org). Flanking sequences of the *mrr* locus (FEK30_09380) were used for recombinant DNA insertion by homologous recombination according to previous literature (Victoria et al. 2024). The synthetic DNA sequences were synthesized by Eurofins Genomics (Edersberg, Germany) as DNA fragments. Two distinct DNA constructs were generated via Gibson Assembly (Thermo Fisher, Waltham, USA). The first construct comprised the *P*_*cpt*_ constitutive promoter (Till et al. 2020), *smr* selection cassette, and the *T*_*7*_ terminator (Supporting Information S1: Figure S1a). The second construct included the *P*_*cpt*_ promoter, *bkt* gene, the *kmr* selection cassette, and the *T*_*7*_ terminator (Supporting Information S1: Figure S1b). The *P*_*cpt*_ promoter and *T*_*7*_ terminator sequences were amplified from plasmids pSW036 (Włodarczyk et al. 2020). Both constructs were cut by *LguI* restriction enzyme (Thermo Fisher Scientific, USA) to facilitate recombination in the target site. The DNA sequences used for genomic transformation (Betterle et al. 2025; Formighieri and Melis 2014; Eaton‐Rye 2011) are available in the Supplementary Information.

### 2.3 Generation of Recombinant Constructs in *Synechocystis*

The recombinant plasmid pTest_kan used for the transformation of *Synechocystis* derives from plasmid pSuperP_UV (Loprete et al. 2025) from which the promoter sequence *P*_*cpc560*_ was excised with *BglII* and *BamHI* restriction enzymes. The re‐ligated plasmid maintained the terminator sequence *T*_*rbc*_ and the *kmr* resistance cassette, flanked by regions ns5' and ns3' (nucleotides 156,282–156,982 and 157,030–157,496, respectively) (Loprete et al. 2025) (Supporting Information S1: Figure S1c). After purification, the construct was cut by *DraI* restriction enzyme. The DNA sequences used for genomic transformation are available in the Supplementary Information.

### 2.4 Conventional Transformation of *Picosynechococcus*

The transformation procedure required the addition of 0.5–1 µg of plasmid DNA to 1 mL of culture at an optical density (OD_730_) (Jasco V‐730) of approximately 0.8. The mixture was then incubated overnight at 30°C under continuous exposure to warm white LED light (150 µmol photons/m²/s), shaking at 120 rpm. Subsequently, the cells were plated onto solid medium containing the appropriate antibiotic and incubated for 7 days. The emerging colonies were then re‐streaked several times to ensure complete segregation, according to previous literature (Victoria et al. 2024; Włodarczyk et al. 2020). Due to the randomic nature of clone selection and characterization of mutants in plates, a direct comparison between the timing of this method and the CSTR‐based one is not possible, as they are completely different approaches of cultivation. A further discussion is reported later in the manuscript.

### 2.5 Selection and Cultivation of Transformants in the CSTR System

**Figure 1:**
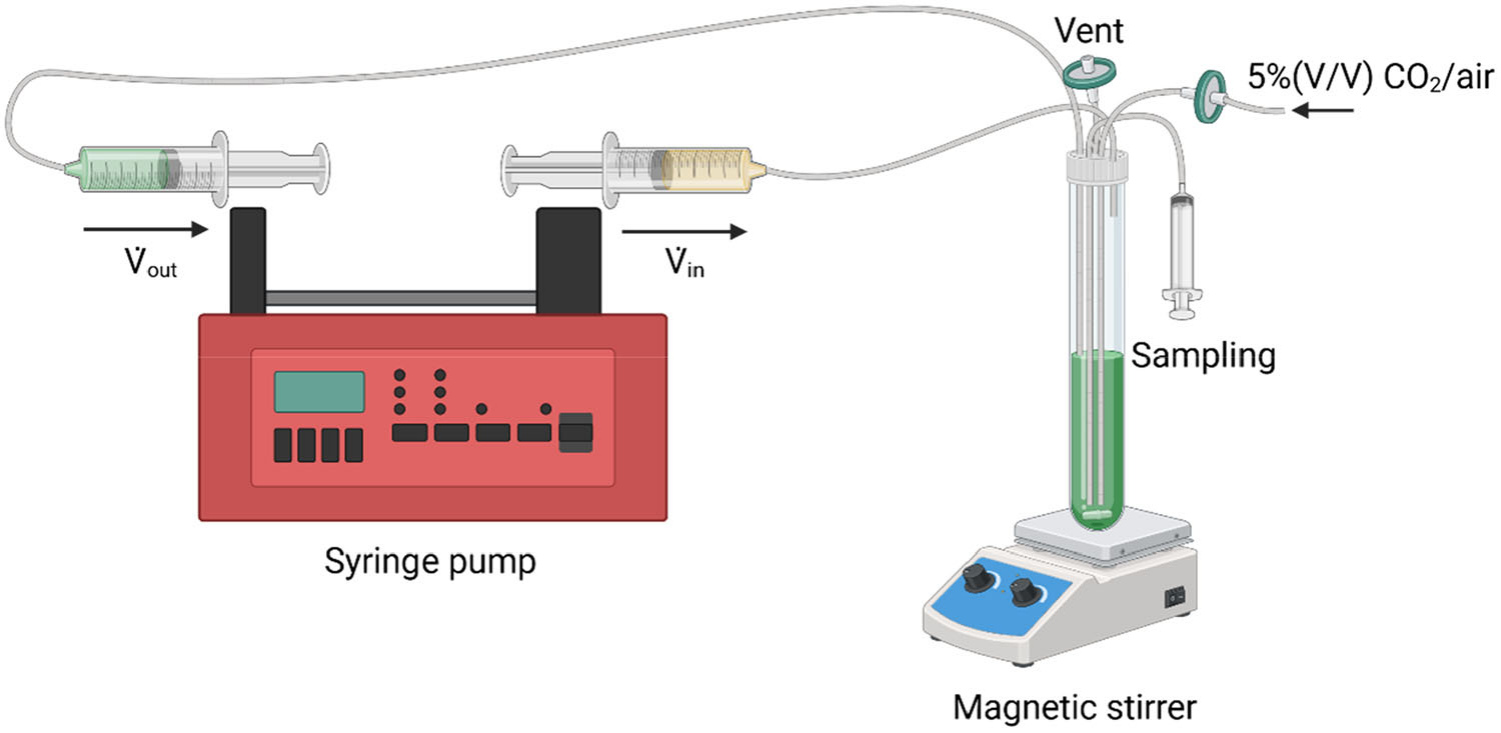
Schematic representation of the cultivation system with inlet and outlet being controlled by a syringe pump; tubular millireactor provided with vent, sampling, air inlet, medium inlet and outlet. Mixing is ensured by a magnetic stirrer. Created in BioRender. Salvagnini, C. (2025) https://BioRender.com/q49q125.

Transformation and selection experiments were conducted in small cylindrical glass photobioreactors, referred to as “millireactors,” with dimensions of 2.3 cm in diameter and 15 cm in height, previously sterilized by autoclaving. Mixing was ensured by a magnetic stirrer. The polycarbonate top of the reactors was equipped with five silicone tubes that ensure input of fresh medium ($\dot{V}$_in_), output of biomass and spent medium ($\dot{V}$_out_), sampling, input of CO_2_ and venting, as shown in Figure 1. The flows through the reactor were regulated by a syringe pump (Harvard Apparatus PHD ULTRATM). The input medium and waste material were contained in 50 mL syringes, appropriately positioned on the pump and connected to the reactor via silicone tubing. Equal inlet and outlet flow rates were maintained to ensure a constant reactor volume, forming a closed CSTR system. In such a system, *τ* (*d*) represents the residence time, that is, the average time a fluid spends inside the reactor and is calculated as $\tau =\frac{V_{R}}{\dot{V}}$ where *V_R_* (*L*) is the volume of the reactor and $\dot{V}$ (*L*/*d*) is the inlet flow (Peng 1994). Residence time can also be described as the inverse of dilution rate (D), which, in a CSTR, is equal to the specific growth rate *µ* (*d* ^−^^1^), therefore $\tau =\frac{1}{D}=\frac{1}{µ}$ (Fernandes et al. 2015). Based on these equivalences, altering the residence time by adjusting either *V_R_* or $\dot{V}$ imposes a different growth rate on the culture. After a transient period, the system reaches a state of equilibrium, called steady state, where biomass concentration and composition remain constant, unless experimental conditions change (Trentin et al. 2023). When τ becomes shorter than the inverse of the growth rate, cells are washed out of the reactor (Ruiz et al. 2013), that is, they do not duplicate quickly enough to remain in the reactor.

### 2.6 Modified Protocol for Natural Transformation and Selection of *Picosynechococcus*

**Figure 2:**
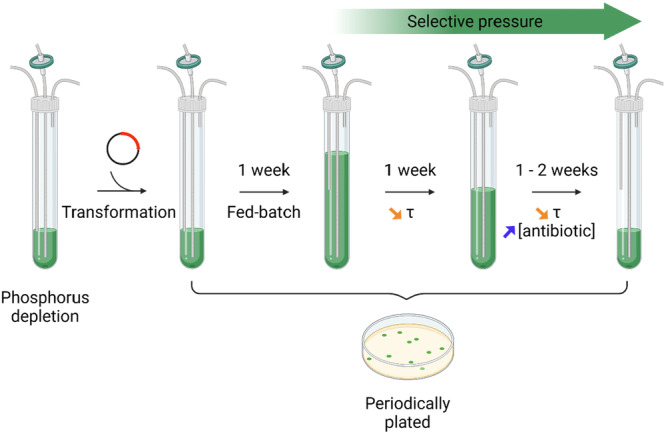
Schematic representation of the transformation and selection process with increase of antibiotic/decrease of residence time over time. Created in BioRender. Salvagnini, C. (2025) https://BioRender.com/x28s424.

**Figure 3:**
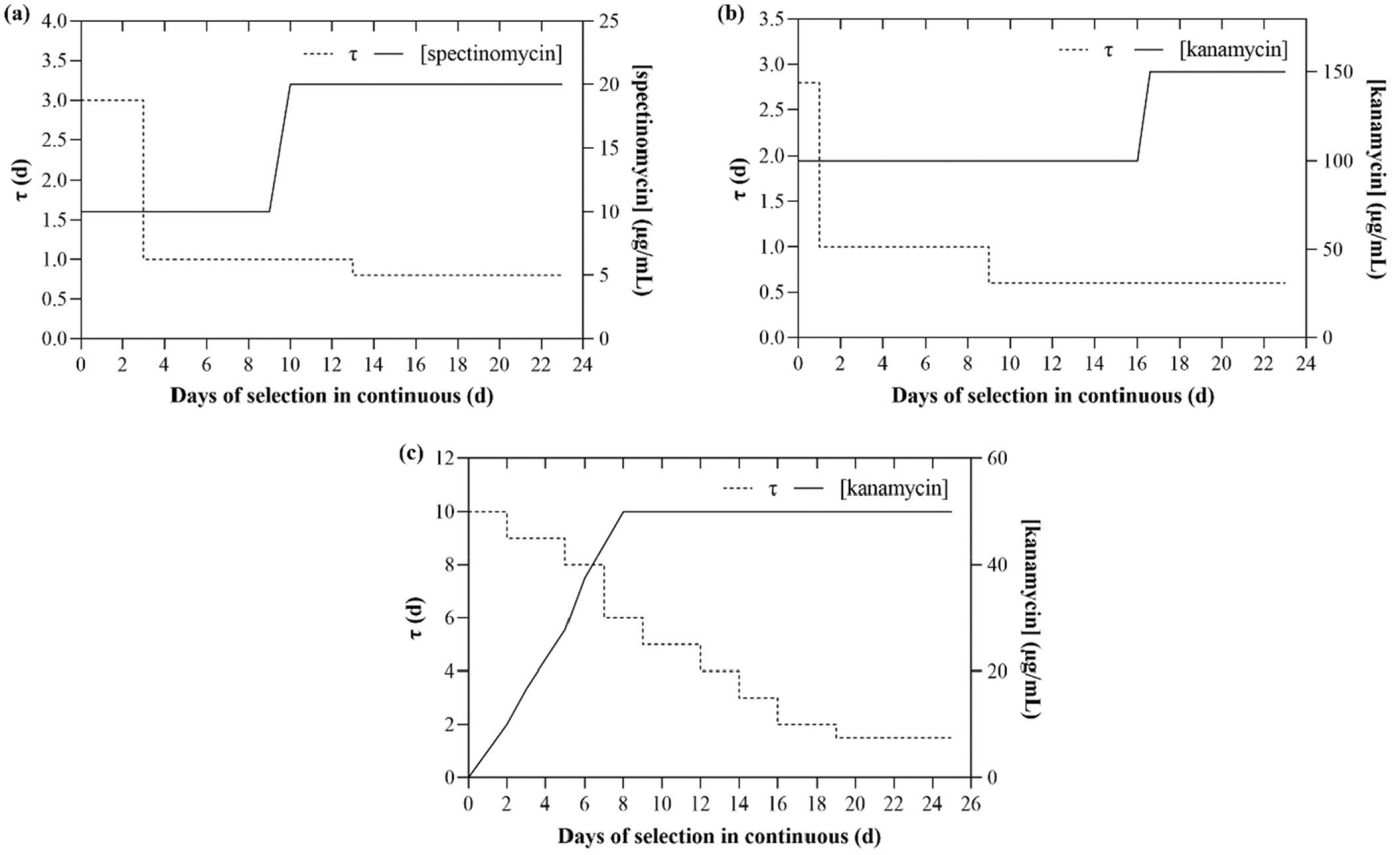
Variation of τ and antibiotic concentration as a function of day of cultivation in continuous during selection of (a) SmR and (b) bKT transformants of *Picosynechococcus*; (c) KmR transformants of *Synechocystis*.

*Picosynechococcus* was grown photo‐autotrophically in a continuous system (*V_R_* = 18.5 mL; *τ* = 1 d) with MAD_low P_ for 48 h. After 2 days of adaptation, an adequate aliquot of culture was resuspended in MAD_low P_ to a final OD_750_ of c.a. 1.5 (dry weight c.a. 1.2 g/L) in a volume of 5 mL. Dry weight (DW) was measured by vacuum‐filtering 5 mL of sample through a pre‐dried cellulose nitrate filter (0.2 μm pore size). After oven‐drying the filter for 2 h, it was weighted and DW was calculated as $\mathrm{DW}=\frac{\mathrm{Grossweig}ht-\mathrm{tare}}{V_{\mathrm{sample}}}$. Approximately 5 µg of DNA were directly added inside of the reactor and natural transformation was allowed to occur overnight in a batch system at 35°C, under stirring, white light LED panel (~ 25 µmol photons/m²/s), 5% (V/V) CO_2_/air bubbling. After the incubation time, 500 µL of culture were withdrawn, pelleted at 13,500 g for 10 min, resuspended in 100 µL of fresh medium and plated on MAD_low P_ petri dishes with 10 µg/mL spectinomycin or 100 µg/mL kanamycin. They were then left in an incubator at 30°C, atmospheric CO_2_, white light LED panel (~ 150 µmol photons/m²/s) until colonies appeared, then transferred at room temperature as a control and backup. The remaining volume of the culture was then slowly diluted in MAD_low P_ with spectinomycin 10 µg/mL or kanamycin 100 µg/mL to a final volume that allows growth without washing out. When the final volume was reached, continuous growth started. For the selection, a similar approach to serial re‐streaking was applied, that is, the increase of antibiotic concentration over time, to emphasize selection. Selective pressure was increased only when steady state was reached in order not to wash out the culture from the reactor. The antibiotic concentrations used for selection are reported in the literature and are commonly used for the tested species in selective petri dishes. The selection process lasted for approximately 3 weeks and is schematically represented in Figure 2. Residence time and antibiotic concentration served as selective factors; the former was gradually shortened, and the latter was increased, as shown in Figure 3a,b. To verify the achievement of homoplasmy in the transformed culture, samples were withdrawn periodically directly from the bioreactor. Subsequently, a PCR analysis was conducted to verify the presence of the construct of interest and of the residual WT sequences, as described in Supplementary Information. *Picosynechococcus* transformants will be hereafter referred to as SmR and bKT.

### 2.7 Modified Protocol for Natural Transformation and Selection of *Synechocystis*

*Synechocystis* was pre‐adapted in BG11_lowP_. Before the transformation, the culture was centrifuged (3000 g for 5 min at room temperature), and the pellet was resuspended in an appropriate volume of BG11_low P_ to achieve a final cell density of 10^10^ cells/mL. 200 μL of culture were transferred into sterile tubes, along with 600 ng of DNA. Samples were incubated at 30°C for 5 h under continuous white LED light (20 µmol photons/m²/s) and stirring (Zang et al. 2007). The culture was then diluted in 10 mL of BG11_low P_, transferred to a millireactor and grown in a batch system with 5% (V/V) CO_2_/air at 85 µmol photons/m²/s. After 24 h, continuous growth began with an inlet of 1 mL/day of fresh medium BG11_low P_ containing 50 µg/mL. The initial residence time (*τ* = 10 days) was decreased over time, maintaining a stable $\dot{V}$ of 1 mL/d and periodically withdrawing 1 mL to decrease V_R_, until a final *τ* = 1.5 day was maintained. Kanamycin concentration was determined based on the cumulative replacement of culture with fresh medium containing 50 µg/mL of antibiotic. The calculation is based on the equation *C_i_V_i_* = *C_f_V_f_*, also accounting for the residual antibiotic concentration already present in the reactor from the previous steps. In the equation, *C_i_* represents the concentration of kanamycin in the fresh inlet medium, *V_f_* is the volume of the reactor (*V_R_*), and *V_i_* is the volume of fresh medium added during the time interval considered. The profile of the decrease of τ and the increase of antibiotic over time is shown in Figure 3c. PCR analysis was conducted to verify the achievement of homoplasmy, as described in Supplementary Information. *Synechocystis* transformants will be hereafter referred to as KmR.

## 3 Results and Discussion

### 3.1 Transformation and Selection of *Picosynechococcus* sp. PCC 11901 With SmR Cassette

**Figure 4:**
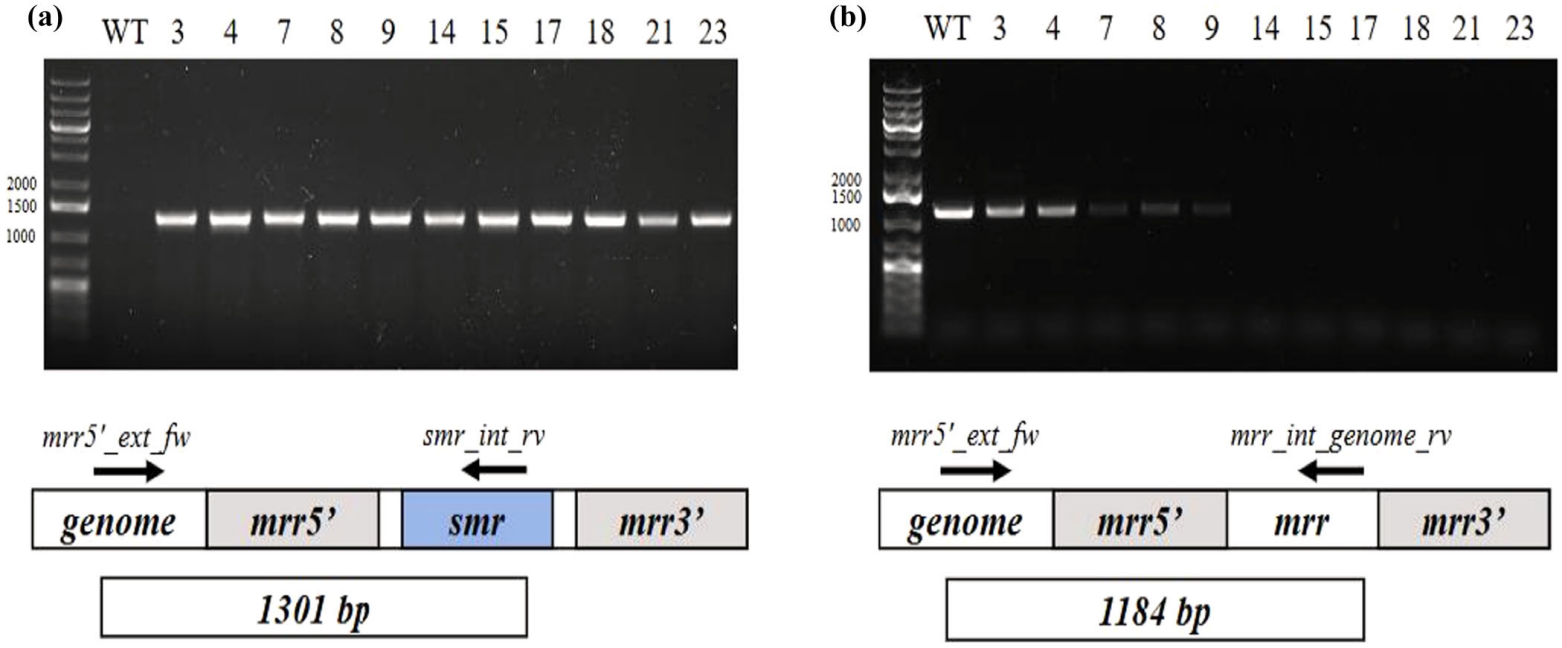
Genomic DNA PCR analyses testing for presence of the transgenic (a) or wild‐type (b) DNA sequences upon 3–23 days of selection in a continuous system of SmR *Picosynechococcus* transformants. Each PCR analysis comprised 35 cycles of DNA amplification, and the expected size of the PCR products was 1301 bp and 1184 bp for the transgenic (primers mrr5'_ext_fw and smr_int_rv) and WT (primers mrr5'_ext_fw and mrr_int_genome_rv) sequences, respectively.

The first transformation experiment involved the insertion of a relatively simple construct containing solely a spectinomycin‐resistance cassette (*smr*) into the *mrr* locus of the genome of *Picosynechococcus* (Supporting Information S1: Figure S1). The *mrr* locus encodes for a putative Type IV restriction endonuclease, which appears to be redundant and was recently identified as a neutral genomic integration site (Victoria et al. 2024). Transformation was conducted following the modified protocol described above. Upon overnight incubation, part of the transformation broth was plated on the selection medium, and several antibiotic‐resistant colonies appeared after approximately 1 week (Supporting Information S1: Figure S2a). The remaining part of the overnight batch was cultivated in a fed‐batch mode, by inletting medium until the final desired volume to start selection in a continuous system was reached. The continuous selection lasted for a total of 23 days, decreasing residence time and increasing spectinomycin concentration as selective pressure factors (Figure 3a) when optical density appeared stable. To track the selection process, the culture was sampled periodically. PCR analyses were performed on DNA directly extracted from liquid culture using two sets of primers appropriately designed to amplify, respectively, a 1301 bp region of the recombinant construct SmR (one primer internal to the resistance cassette and one external to the flanking sequence required for homologous recombination) and a 1184 bp region of the wild‐type genomic sequence (one primer internal to the insertion site and one external to the gene locus). Samples were collected at different timepoints (i.e., days 3, 4, 7, etc.), indicating the day of selection in continuous. The results, illustrated in Figure 4a, show that SmR construct was present in all samples throughout the selection process, confirming the correct integration of the sequence in the host genome. Interestingly, Figure 4b shows that the wild‐type genome, initially present in the mixture, was progressively washed out of the reactor during the days of operation. By Day 14, wild‐type genomes were completely absent, indicating the successful enrichment of homoplasmic transformants in the reactor. This timeline is consistent with, or even more convenient than, achieving full segregation of transformants after two colony re‐streaks, which is not always achievable and is susceptible to random occurrences. Also, working with an automated continuous reactor has the added benefit of reducing the workload of the operator (Mills et al. 2022). Although a direct comparison between the conventional method and the approach proposed here is complicated by the stochastic nature of the transformation process, it is important to note that a single liquid sample taken from a chemostat is representative of the entire population. Therefore, any residual heteroplasmy can be reliably detected by PCR: if no heteroplasmy is detected, it is reasonable to assume that all the cells retained in the reactor have been successfully transformed and attained transgenic DNA copy homoplasmy. Thus, any plating after this point would result in 100% homoplasmic colonies appearing in Petri dishes.

As stated before, cyanobacteria possess multiple chromosome copies within each cell and, for a successful transformation of the transformants, all copies must incorporate the exogenous DNA. To facilitate *Picosynechococcus* transformation, cells were grown in phosphorus‐depleted medium, aiming to minimize the chromosome copy number. This strategy was inspired by a previous work by Pope et al. (2020) on *Synechocystis*, where the phosphorus depletion reduced the median chromosome copy number, facilitating the obtainment of homoplasmic transformants. To further improve transformation efficiency, CO_2_ was directly bubbled into the culture medium, relying on a prior study where a CO_2_‐enriched chamber was employed in the transformation of *Picosynechococcus* (Victoria et al. 2024), a resource unavailable in the present study.

Traditionally, after transformation, fully segregated transformants are selected by repeatedly streaking colonies on solid media containing progressively higher concentrations of antibiotics. The present study proposes an alternative protocol, exploiting the intrinsically selective environment of a CSTR system. In a CSTR, the residence time can be controlled to impose a specific growth rate on the cells. By reducing the residence time, cells are forced to adapt to the faster growth rates required to remain in the reactor (Sharma and Arya 2017). When combined with antibiotic selection, this approach creates conditions that strongly favor antibiotic‐resistant cells capable of growing at the imposed rates, while non‐adapted populations are washed out. Although the population in a chemostat is not entirely homogeneous—as variations in cell size, cell cycle stage, and specific growth rate can be observed—prolonged selective pressure will eventually lead to the washout of cells that lack the advantageous traits. The concept of accelerating evolutionary adaptation exploiting a continuous system is not new (Gresham and Dunham 2014). CSTR systems have been previously applied in reactors to enrich desired populations in mixed cultures by modulating τ (Carrillo‐Reyes et al. 2016; Devlin and Oleszkiewicz 2018). However, to the best of our knowledge, no protocol has yet been reported for selecting transformant cyanobacteria in a CSTR system with the purpose of segregating homoplasmic strains. In the initial stages of selection, two populations coexist in the reactor: a small number of transformed cells, essentially heteroplasmic, and a larger population of non‐transformed, wild‐type cells. When these populations are subjected to antibiotic selection, the nontransformed cells cannot replicate and are eventually washed out of the reactor. Over time, homoplasmic cells ‐ containing exclusively transformed chromosomes ‐ outcompete heteroplasmic cells, due to their higher resistance to the antibiotic, as demonstrated by the complete loss of wild‐type DNA after 2 weeks of selection (Figure 4b).

### 3.2 Transformation and Selection of *Picosynechococcus* With bKT Construct

The second transformation experiment involved a more complex construct carrying a β‐carotene 4‐ketolase gene (*bkt*) and a kanamycin resistance cassette (*kmr*) (Supporting Information S1: Figure S1b). The expression of *bkt* gene induces a significant rearrangement in pigment profile, imposing a physiological burden on phototrophic cells (Betterle et al. 2025), rendering the transformation more challenging. The construct was successfully integrated into the *mrr* locus and several antibiotic‐resistant colonies appeared on selective plates (Supporting Information S1: Figure S2b). The selection process in the CSTR system was conducted by reducing τ and increasing kanamycin as selective agents, as shown in Figure 3b. The higher concentration of kanamycin with respect to spectinomycin adopted in this study was chosen accordingly to previous literature (Victoria et al. 2024; Włodarczyk et al. 2020). Absorption spectra of chlorophyll and carotenoids extracted from cells sampled during the selection in CSTR revealed a shoulder between 500 and 550 nm in bKT transformants that was absent in WT samples (Supporting Information S1: Figure S3a). Such shoulder is attributable to the accumulation of ketocarotenoid, as according to literature (Betterle et al. 2025; Perozeni et al. 2020; Shimada et al. 2020; Hasunuma et al. 2019). Thin layer chromatography (TLC) analysis evidenced a new orange‐pigmented band in the extract from the transformed cells as the most abundant carotenoid (Supporting Information S1: Figure S3b), attributable to canthaxanthin according to literature (Betterle et al. 2025). Protocols used for absorption spectra analysis and TLC are described in Supplementary Information.

**Figure 5:**
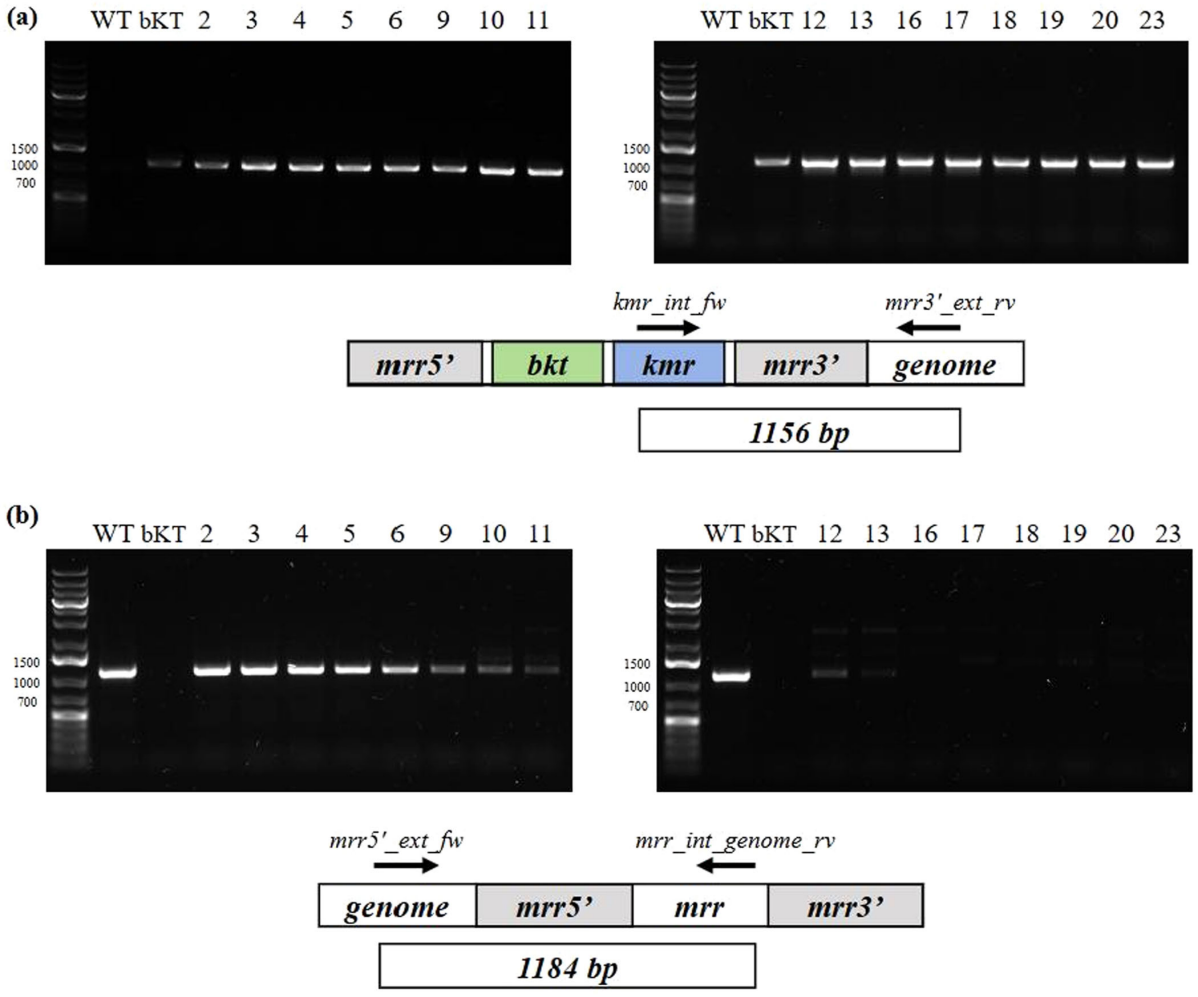
Genomic DNA PCR analyses testing for the presence of the transgenic (a) or wild‐type (b) DNA sequences upon 2–23 days of selection in a continuous system of bKT *Picosynechococcus* transformants. Each PCR analysis comprised 35 cycles of DNA amplification, and the expected size of the PCR products was 1156 bp and 1184 bp for the transgenic (primers kmr_int_fw and mrr3'_ext_rv) and WT (primers mrr5'_ext_fw and mrr_int_genome_rv) sequences, respectively. A bKT transformant, already available in the laboratory and obtained through conventional transformation, was used as a positive control for the presence of *bkt*.

Primers were designed to amplify a 1156 bp region of the recombinant bKT construct and a 1184 bp region of the wild‐type genome. PCR analyses showed the presence of bKT construct throughout the whole selection process (Figure 5a). On the other hand, wild‐type DNA was progressively eliminated within 16 days of selection (Figure 5b). This confirms the observation made in the previous section, suggesting that the time needed to wash out the heteroplasmic cells is approximately the same, even with different selection agents and lengths of the construct. However, as reported in Figure 5b, it is clear that after 9 days, the number of WT genome copies is underrepresented, suggesting that the percentage of homoplasmic transformants is increasing quickly.

### 3.3 Transformants Stability in a Semi‐Continuous System

**Figure 6:**
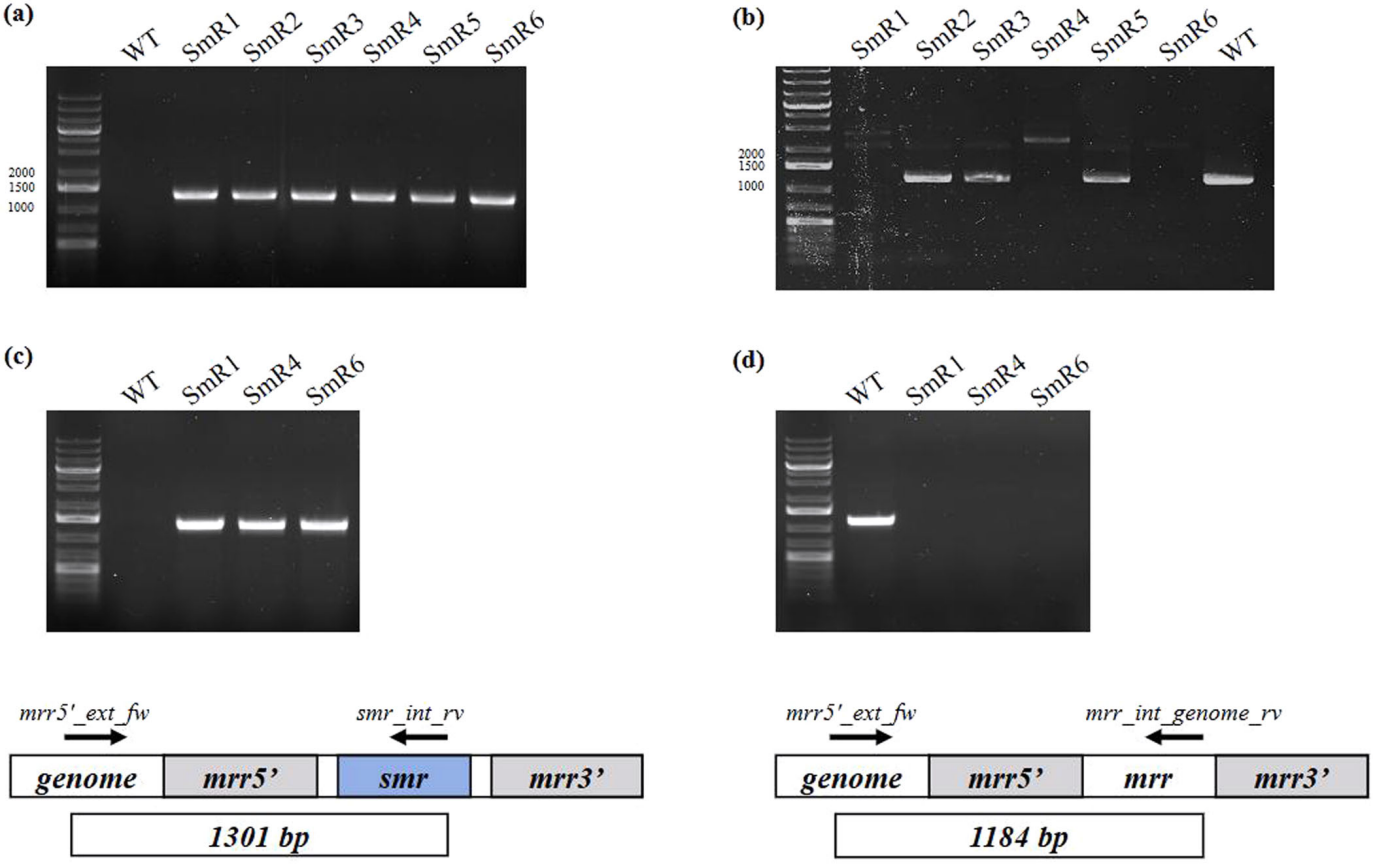
Genomic DNA PCR analyses testing for presence of the transgenic (a) or wild‐type (b) DNA sequences on colonies SmR1‐6 of Picosynechococcus after three weeks of batch culture; Genomic DNA PCR analyses testing for presence of the transgenic (c) or wild‐type (d) DNA sequences on colonies SmR1, SmR4 and SmR6 after 6 weeks of growth in a semi‐continuous system (*τ* = 4 days). Each PCR analysis comprised 35 cycles of DNA amplification, and the expected size of the PCR products was 1301 bp and 1184 bp for the transgenic (primers mrr5'_ext_fw and smr_int_rv) and WT (primers mrr5'_ext_fw and mrr_int_genome_rv) sequences, respectively.

After 3 days of selection in continuous of the SmR transformant of *Picosynechococcus*, an aliquot of liquid culture was plated onto solid medium, as described in the Supplementary Information. Once visible colonies appeared, six were randomly picked and named SmR1 through SmR6. These colonies were transferred into fresh liquid medium that lacked spectinomycin to allow growth without selective pressure. Colonies were firstly cultivated in a batch system for 3 weeks to produce sufficient biomass for PCR analysis. PCR assays were conducted on all six colonies to determine whether they had achieved homoplasmy. The results, presented in Figure 6a, confirmed that all colonies contained the construct of interest. Furthermore, three colonies, SmR1, SmR4 and SmR6 were identified as homoplasmic based on the complete absence of WT (Figure 6b). To investigate the stability of the homoplasmic transformants under prolonged cultivation in the absence of antibiotics, SmR1, SmR4 and SmR6 were subsequently grown in a semi‐continuous system with a *τ* = 4 days. The cultivation was carried out for 6 weeks. The results, presented in Figure 6c,d, demonstrated that SmR1, SmR4 and SmR6 successfully maintained the construct of interest and retained their homoplasmic status throughout the whole experiment, over the course of 42 days of cultivation in semi‐continuous without spectinomycin. Growth was monitored via OD_750_ and is shown in Supporting Information S1: Figure S4.

### 3.4 Validation of Selection Protocol on the Model Species *Synechocystis*

The transformation of *Synechocystis* involved the insertion of a construct containing a kanamycin‐resistance cassette into a neutral site (*ns*) of the genome (Supporting Information S1: Figure S1c). The *ns* is a region downstream of the NADH dehydrogenase gene (*ndhb*) and interrupts the ORF ssl0410 that codes for an unknown putative protein of 90 amino acids; it is a well‐known site for transformation of *Synechocystis* (Ohkawa et al. 2000; Pinto et al. 2015; Loprete et al. 2025). The adjustments made to residence time and antibiotic concentration over the course of selection are summarized in Figure 3c. To assess the homoplasmy of KmR transformants, PCR analysis was performed using primers listed in Supporting Information S1: Table S1. Two primers, designed on the flanking regions ns5' and ns3', were selected to amplify two distinct sequences: a 2056 bp region on the transformed genome and a 617 bp region of the wild‐type genome. The presence of two bands would indicate a heteroplasmic population, while a single band at 2056 bp would confirm the presence of homoplasmic cells. The results, shown in Supporting Information S1: Figure S5, revealed that from Day 1 to 19 of the selection process, both the 2056 bp and 617 bp bands were present, indicating that the population was still heteroplasmic. However, by Day 21, only the 2056 bp band remained, confirming that cells containing wild‐type DNA had been entirely washed out of the reactor.

*Synechocystis* was the first phototrophic organism whose genome was fully sequenced, making it a model species for cyanobacteria studies (Ikeuchi and Tabata 2001). This cyanobacterial strain is known to contain a large number of chromosome copies ‐ depending on growth phase (Zerulla et al. 2016) ‐ presenting a significant challenge when attempting to select fully segregated transformants. To determine whether the proposed selection protocol could be applied successfully to other cyanobacteria, it was tested on this model species. Once again, wild‐type chromosomes were entirely washed out of the reactor in under 21 days of selection in continuous, leading to the successful obtainment of fully segregated transformants. Compared to *Picosynechococcus*, where transformant selection was achieved in 2 weeks, the longer selection duration for *Synechocystis* may be attributed to the higher number of chromosome copies present in the latter organism. Further studies should focus on a direct side‐by‐side comparison of conventional and CSTR‐based selection for transformants, with particular attention to the number of generations required to achieve full segregation. Also, the efficacy of the method on multiple site insertions should be validated. It should be noticed, anyway, that the number of generations is generally much higher in a continuous system, where cells are naturally forced to maintain the exponential phase of growth. This could be an advantage for gene integrations and selection of stronger clones, without impairments of the growth, which would result in a washout from the system. As an example, in this study we found that transformation of *Picosynechoccus* with SmR construct required approximately 17 generations to reach full segregation, whereas bKT construct required about 28 generations. It is important to note that different antibiotics were used in these selection protocols, which are differently tolerated by the cyanobacteria (Victoria et al. 2024) and may have influenced the number of generations needed. Additionally, the bKT construct involves ketocarotenoids accumulation, resulting in a significant rearrangement of pigment profile (Betterle et al. 2025), which could have contributed to a slower segregation progress. For *Synechocystis*, complete transformant segregation required 8.2 generations. The results of the present study demonstrate a proof of concept that the selection protocol in a CSTR system successfully enriches for fully segregated transformants in *Picosynechococcus* and *Synechocystis*, highlighting its potential applicability to other cyanobacterial species with different ranges of chromosome copy number.

## 4 Conclusions

This study presents a novel protocol for the transformation and selection of homoplasmic transformants in *Picosynechococcus*, overcoming the key challenge of polyploidy in cyanobacterial genetic engineering. The proposed system of selection in a CSTR introduces dual selective pressure, combining antibiotic resistance and growth rate constraints imposed by residence time. This system effectively favors homoplasmic transformants while progressively eliminating heteroplasmic cells.

The success of the method was demonstrated through the transformation of *Picosynechococcus* with two constructs of increasing complexity ‐ SmR and bKT ‐ and achieving the selection of fully segregated transformants in a CSTR system, with a rapid elimination of heteroplasmic cells within 14–16 days. The impact of this protocol is linked to the ease of the procedure, avoiding the efforts required to sequentially re‐streak single colonies, a time‐consuming procedure that only selects for a few homoplasmic colonies at a time. On the other hand, this method allows for the selection of a transgenic population of cells that is already adapted to growth in a continuous system, making it a great potential for engineering strains exploitable in industrial cultivation. Additionally, the stability of single colonies of SmR transformants under prolonged cultivation without antibiotic pressure further suggests the suitability of such selected transformants for industrial applicability.

Finally, the versatility of this protocol was demonstrated by its successful validation on *Synechocystis*, a model cyanobacterium containing up to several dozen chromosome copies, showing great potential for broader applications across different cyanobacterial strains.

## Author Contributions

**Cecilia Salvagnini:** investigation (equal), writing – original draft preparation (lead), visualization (lead), data curation. **Eliana Gasparotto:** investigation (equal), writing – original draft preparation (supporting), visualization (supporting). **Veronica Lucato:** investigation (supporting), writing – original draft preparation (supporting), visualization (supporting), validation. **Elisabetta Bergantino:** writing – review and editing. **Matteo Ballottari:** writing – review and editing. **Elena Barbera:** writing – review and editing, funding. **Nico Betterle:** supervision, writing – review and editing, funding. **Eleonora Sforza:** conceptualization, supervision, writing – review and editing, project administration.

## Supporting information

Revised Supplementary Salvagnini et al.

## Data Availability

The data that support the findings of this study are available from the corresponding author upon reasonable request.
