# Supplementary material for: A Bioprocess Engineering Approach to Boost Selection of Fully Segregated Transformants in Cyanobacteria
Source: Biotechnol Bioeng. 2025 Jul 14;122(10):2781–90. doi: 10.1002/bit.70024 (PMC12417791; doi:10.1002/bit.70024)
Supplement: Supplementary file 1 — Revised Supplementary Salvagnini et al. [file BIT-122-2781-s001.docx]

**A bioprocess engineering approach to boost selection of fully segregated transformants in cyanobacteria**

Cecilia Salvagnini^a^, Eliana Gasparotto^b^, Veronica Lucato^a^, Elisabetta Bergantino^c^, Matteo Ballottari^b^, Elena Barbera^a^, Nico Betterle^b^ and Eleonora Sforza^a^

*^a^Department of Industrial Engineering, University of Padova*

*^b^Department of Biotechnology, University of Verona*

*^c^Department of Biology, University of Padova*

**Short title**: Enhancing mutant selection in cyanobacteria

SUPPLEMENTARY INFORMATION

Submitted to Biotechnology and Bioengineering

Corresponding author:

Eleonora Sforza,

Via Marzolo 9,

35131 Padova (PD),

[eleonora.sforza@unipd.it](mailto:eleonora.sforza@unipd.it) +39 049 827 5467

**SUPPLEMENTARY MATERIALS AND METHODS**

**Constructs for genomic transformation**

For the transformation of *Picosynechococcus*, constructs SmR and bKT were designed as illustrated in **Figure** **S1a** and **S1b**. Construct KmR (**Figure S1c**) was used for the transformation of *Synechocystis*.


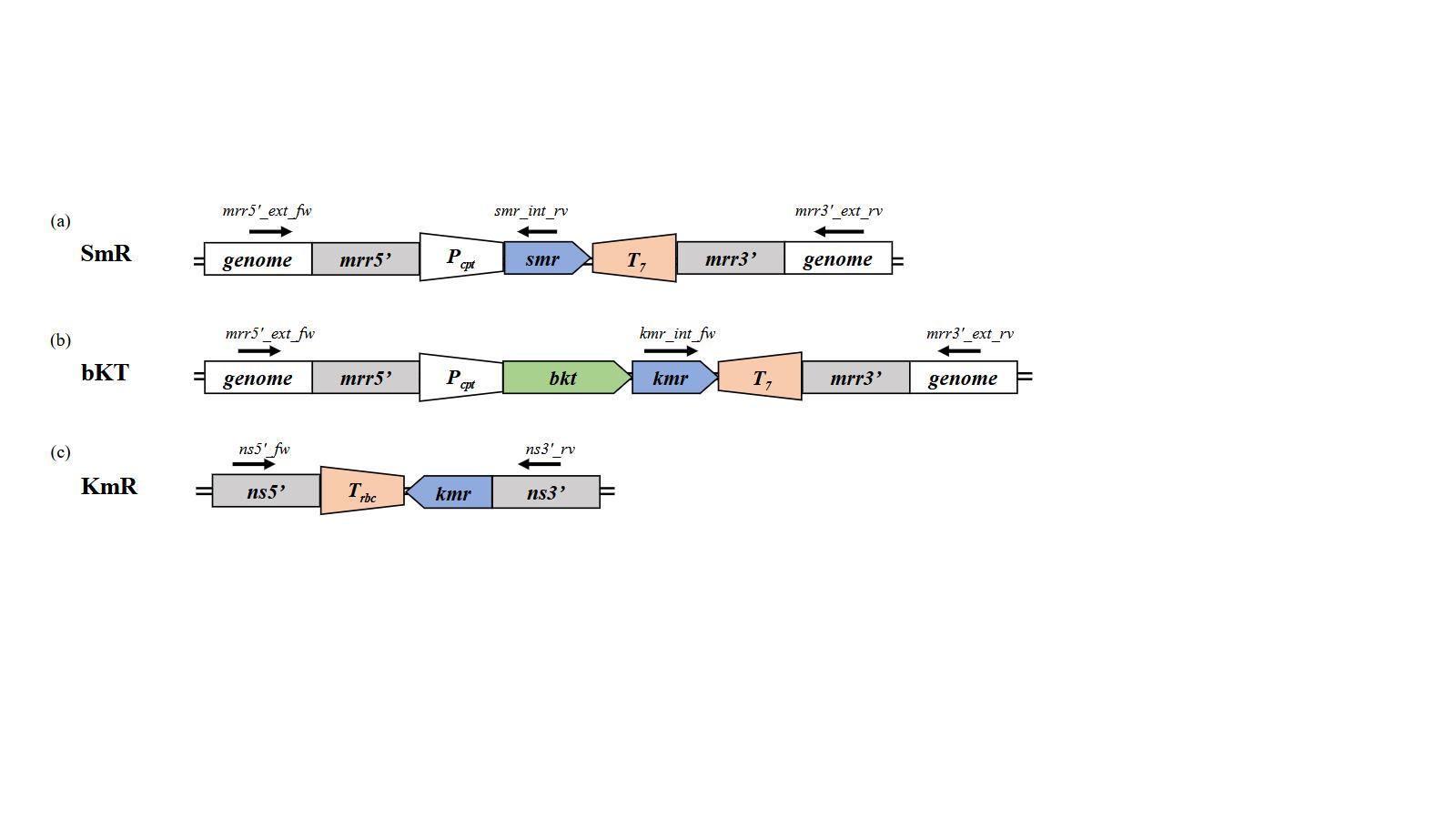
**Figure S1**: Schematic representation of constructs **(a)** SmR for the transformation of *Picosynechococcus*, carrying the *P_cpt_* promoter, a spectinomycin resistance cassette (*smr*), and the *T_7_* terminator, flanked by regions *mrr*5’ and *mrr*3’ used for homologous recombination; **(b)** bKT for the transformation of *Picosynechococcus*, carrying the *P_cpt_* promoter, a codon-optimized *β-carotene ketolase* gene (*bkt*) from *Chlamydomonas reinhardtii*, a kanamycin-resistance cassette (*kmr*), and the *T_7_* terminator flanked by regions *mrr*5’ and *mrr*3’ used for homologous recombination; **(c)** KmR for the transformation of *Synechocystis*, carrying a kanamycin-resistance cassette (*kmr*) and the *T_rbc_* terminator, flanked by regions *ns*5’ and *ns3*’ used for homologous recombination. Arrows mark the position of oligonucleotide primers used for genomic DNA PCR analysis.

**DNA sequences for genomic transformation**

- **SmR construct sequence**: Flanking 5’, *P_cpt_* promoter, *smr* antibiotic resistance, *T_7_* terminator, Flanking 3’

caatcaatacagctatggccgcaatgataagggtcaggcacgcccggaggtcttgaaaaaattactccaaacaggcgatcgcctcgtgcaagaaattgactccgtggaatatggcctgacagatatccaggaatactacgccaataccggggggctgaaaaaagcggcggaaaatgccagcggtaaaaccgttacggccagctttgtggaaagtttctcgaaggacacaacgccccgaaaattagaatcggtgctgcgcatggaatatcgctctaagctgctcaaccccaagtgggctgaggcaatggccgatcaggggtctgggggagcctatgaaatttcccagcgaatgacagctttaatcggttggggcggtacagtcgatttccaggatcaatgggtctatgaccaagccgcagagacctatgcccttgatgcagccatggcgcaaaaattgcgagaggccaaccccgaagctttccggaatattgtcgggcgaatgttggaagcccatgggcgcggtttctgggaagcttccgatgaaaaattgcaaaaactaagtgatctttatgaccttacggacgatgttttagaaggaattacaacttaattgagacaagatttcaaagatttgtaagaagcttaatcccttaagatttagggtgttgatcatggatatagaaattagcacacgctttttttaacaaaaaagcaggaataaaattaacaagatgtaacagacataagtcccatcaccgttgtataaagttaactgtgggattgcaaaaggagctcccaactcataaagtcaagtaggagattaattccATGAGGGAAGCGGTGATCGCCGAAGTATCGACTCAACTATCAGAGGTAGTTGGCGTCATCGAGCGCCATCTCGAACCGACGTTGCTGGCCGTACATTTGTACGGCTCCGCAGTGGATGGCGGCCTGAAGCCACACAGTGATATTGATTTGCTGGTTACGGTGACCGTAAGGCTTGATGAAACAACGCGGCGAGCTTTGATCAACGACCTTTTGGAAACTTCGGCTTCCCCTGGAGAGAGCGAGATTCTCCGCGCTGTAGAAGTCACCATTGTTGTGCACGACGACATCATTCCGTGGCGTTATCCAGCTAAGCGCGAACTGCAATTTGGAGAATGGCAGCGCAATGACATTCTTGCAGGTATCTTCGAGCCAGCCACGATCGACATTGATCTGGCTATCTTGCTGACAAAAGCAAGAGAACATAGCGTTGCCTTGGTAGGTCCAGCGGCGGAGGAACTCTTTGATCCGGTTCCTGAACAGGATCTATTTGAGGCGCTAAATGAAACCTTAACGCTATGGAACTCGCCGCCCGACTGGGCTGGCGATGAGCGAAATGTAGTGCTTACGTTGTCCCGCATTTGGTACAGCGCAGTAACCGGCAAAATCGCGCCGAAGGATGTCGCTGCCGACTGGGCAATGGAGCGCCTGCCGGCCCAGTATCAGCCCGTCATACTTGAAGCTAGACAGGCTTATCTTGGACAAGAAGAAGATCGCTTGGCCTCGCGCGCAGATCAGTTGGAAGAATTTGTCCACTACGTGAAAGGCGAGATCACCAAGGTAGTCGGCAAATAAcgagggcggtgctttggcaggatccggctgctaacaaagcccgaaaggaagctgagttggctgctgccaccgctgagcaataactagcataaccccttggggcctctaaacgggtcttgacgggttttttgtctagatcaacggcctcaattcaagagctagaaattcatccatgttttagttcactaattttatttaattctgctgctggaaatcacgccatggttcgaaaatctgccctaaagaatcttcagacagttcctggggagaaattgtttcccaaggctcttttaaaataccctgatctttgtagggatcttgatctaaacaataggcaattaattcgctacaaatccaagcatctgctttatttaaaagttgagcaatttgcttttccaactgaccctgagataatcgattgagaaaatgaccgagcattgtgccagccgccgcatgggccgcaattaaacgcagatcatattcactattgagttcttgttccgcacggttaatcaagttcgttgcaatatcttcactgagacctcttggttttctgaagaaaatgtcgcactgttcatcgtcaaaatatttacttaaattcgatctttgtactcctgattgtgcatgggcttcaatgcactcatcggctcccgtcacaatcaaggtatgggtcgtgataatatcactcatcctgccaaggcctgtaaagtaggcaatgcctctggaaacgaagtttgagtcgttgtaggtaaaaccaatgtaccctcgattatagttgtctccgtactttggagtccggttttgtttcttagagaccatttataaagtaaatctttaggaaactagccgt

- **bKT construct sequence**: Flanking 5’, *P_cpt_* promoter, *bkt* sequence, *kmr* antibiotic resistance, *T_7_* terminator, Flanking 3’

caatcaatacagctatggccgcaatgataagggtcaggcacgcccggaggtcttgaaaaaattactccaaacaggcgatcgcctcgtgcaagaaattgactccgtggaatatggcctgacagatatccaggaatactacgccaataccggggggctgaaaaaagcggcggaaaatgccagcggtaaaaccgttacggccagctttgtggaaagtttctcgaaggacacaacgccccgaaaattagaatcggtgctgcgcatggaatatcgctctaagctgctcaaccccaagtgggctgaggcaatggccgatcaggggtctgggggagcctatgaaatttcccagcgaatgacagctttaatcggttggggcggtacagtcgatttccaggatcaatgggtctatgaccaagccgcagagacctatgcccttgatgcagccatggcgcaaaaattgcgagaggccaaccccgaagctttccggaatattgtcgggcgaatgttggaagcccatgggcgcggtttctgggaagcttccgatgaaaaattgcaaaaactaagtgatctttatgaccttacggacgatgttttagaaggaattacaacttaattgagacaagatttcaaagatttgtaagaagcttaatcccttaagatttagggtgttgatcatggatatagaaattagcacacgctttttttaacaaaaaagcaggaataaaattaacaagatgtaacagacataagtcccatcaccgttgtataaagttaactgtgggattgcaaaaggagctcccaactcataaagtcaagtaggagattaattccATGGCCGCAGCCCTCACGGCCCGTCGGGTAAAGCAGTTCACAAAACAGTTCCGCAGTCGGCGTATGGCCGAGGACATTTTGAAGCTCTGGCAACGGCAATACCACCTGCCCCGGGAAGATTCTGATAAACGCACGCTCCGTGAACGGGTCCACTTGTACCGCCCCCCGCGCTCGGATCTGGGAGGTATCGCGGTCGCCGTTACTGTCATCGCATTGTGGGCTACTTTGTTTGTATATGGGTTGTGGTTTGTTAAACTCCCCTGGGCTCTCAAAGTCGGAGAAACTGCGACTTCGTGGGCGACGATTGCAGCAGTTTTCTTTTCGTTGGAGTTCTTGTATACAGGTCTGTTCATTACCACGCACGACGCCATGCACGGGACGATCGCACTCCGTAATCGCCGCCTCAATGATTTCCTCGGTCAGCTCGCTATCTCCCTCTACGCCTGGTTTGACTACTCTGTCCTCCATCGCAAGCATTGGGAACACCACAATCACACGGGCGAACCTCGCGTTGATCCAGACTTCCATCGTGGCAATCCCAACTTGGCAGTATGGTTTGCTCAGTTCATGGTAAGCTACATGACATTGAGTCAATTCTTAAAGATCGCGGTTTGGTCCAACTTACTGTTGTTAGCCGGTGCTCCGCTCGCTAATCAGTTACTGTTTATGACTGCGGCACCGATCCTCAGCGCATTTCGGTTGTTTTACTATGGCACGTATGTTCCACACCATCCGGAGAAAGGTCATACAGGGGCCATGCCATGGCAGGTTTCGCGGACTTCGTCGGCAAGTCGGTTGCAGAGTTTTCTGACCTGTTACCATTTTGACCTGCACTGGGAACATCACCGTTGGCCATACGCACCGTGGTGGGAACTCCCAAAGTGTCGGCAGATTGCGCGTGGTGCCGCCTTGGCCcaccatcaccatcaccattaaggaattaggaggtaatatATGAGCCATATTCAACGGGAAACGTCGAGGCCGCGATTAAATTCCAACATGGATGCTGATTTATATGGGTATAAATGGGCTCGCGATAATGTCGGGCAATCAGGTGCGACAATCTATCGCTTGTATGGGAAGCCCGATGCGCCAGAGTTGTTTCTGAAACATGGCAAAGGTAGCGTTGCCAATGATGTTACAGATGAGATGGTCAGACTAAACTGGCTGACGGAATTTATGCCTCTTCCGACCATCAAGCATTTTATCCGTACTCCTGATGATGCATGGTTACTCACCACTGCGATCCCCGGAAAAACAGCATTCCAGGTATTAGAAGAATATCCTGATTCAGGTGAAAATATTGTTGATGCGCTGGCAGTGTTCCTGCGCCGGTTGCATTCGATTCCTGTTTGTAATTGTCCTTTTAACAGCGATCGCGTATTTCGTCTCGCTCAGGCGCAATCACGAATGAATAACGGTTTGGTTGATGCGAGTGATTTTGATGACGAGCGTAATGGCTGGCCTGTTGAACAAGTCTGGAAAGAAATGCATAAACTTTTGCCATTCTCACCGGATTCAGTCGTCACTCATGGTGATTTCTCACTTGATAACCTTATTTTTGACGAGGGGAAATTAATAGGTTGTATTGATGTTGGACGAGTCGGAATCGCAGACCGATACCAGGATCTTGCCATCCTATGGAACTGCCTCGGTGAGTTTTCTCCTTCATTACAGAAACGGCTTTTTCAAAAATATGGTATTGATAATCCTGATATGAATAAATTGCAGTTTCATTTGATGCTCGATGAGTTTTTCTAAcgagggcggtgctttggcaggatccggctgctaacaaagcccgaaaggaagctgagttggctgctgccaccgctgagcaataactagcataaccccttggggcctctaaacgggtcttgacgggttttttgtctagatcaacggcctcaattcaagagctagaaattcatccatgttttagttcactaattttatttaattctgctgctggaaatcacgccatggttcgaaaatctgccctaaagaatcttcagacagttcctggggagaaattgtttcccaaggctcttttaaaataccctgatctttgtagggatcttgatctaaacaataggcaattaattcgctacaaatccaagcatctgctttatttaaaagttgagcaatttgcttttccaactgaccctgagataatcgattgagaaaatgaccgagcattgtgccagccgccgcatgggccgcaattaaacgcagatcatattcactattgagttcttgttccgcacggttaatcaagttcgttgcaatatcttcactgagacctcttggttttctgaagaaaatgtcgcactgttcatcgtcaaaatatttacttaaattcgatctttgtactcctgattgtgcatgggcttcaatgcactcatcggctcccgtcacaatcaaggtatgggtcgtgataatatcactcatcctgccaaggcctgtaaagtaggcaatgcctctggaaacgaagtttgagtcgttgtaggtaaaaccaatgtaccctcgattatagttgtctccgtactttggagtccggttttgtttcttagagaccatttataaagtaaatctttaggaaactagccgt

- **KmR construct sequence**: Flanking 5’, *T_rbc_* terminator, *kmr* antibiotic resistance, Flanking 3’

cggtggtttcccaggggcagagcgtccccgtaagatgagatttttaaagacccccattagcgtggggctatccctttaaaaaccgtctttattctggagaatctcaatgcatagctttttgttggccaccgccgttcccgccaccctgtcctggagccctaaagttgctggggtgatgattgcttgcaacattttggcgatcgcctttggtaaattgaccatcaaacaacaaaatgtgggcacccccatgccttcctctaacttctttggcggctttggtttaggggctgtgctgggcaccgctagctttggccacatcctcggcgctggagtaattctggggctagccaatatgggagtactttaaggctcgattctgaatggactagcttttatcctttgggaaaatatcaaaggcgatcgggcaattgaaagaaaagcctggtcgcttttttgttagggattagggaaaatgccaaaacgcaccaaggtggtaattatggctccgatgacggcaagaatcaacgcccaaatttgagcattagcccgccctttgacatctttaacatcatccttgactgtacctatctccatcctgaccgcagataactcggttttcaccgttgccatatcgatcttaagagaagttacatctttttggaggtcatcgagtttggtcttaatttcccccaagatccgaattcgagctccgtcgacaagcttgcggccgcactcgagcaccaccaccaccaccactgaaccggtgtttggattgtcggagttgtactcgtccgttaaggatgaacagttcttcggggttgagtctgctaactaattagccattaacagcggcttaactaacagttagtcattggcaattgtcaaaaaattgttaatcagccaaaacccactgcttactgatgttcaacttcgacagcctgcaggggggggggggcgctgaggtctgcctcgtgaagaaggtgttgctgactcataccaggcctgaatcgccccatcatccagccagaaagtgagggagccacggttgatgagagctttgttgtaggtggaccagttggtgattttgaacttttgctttgccacggaacggtctgcgttgtcgggaagatgcgtgatctgatccttcaactcagcaaaagttcgatttattcaacaaagccgccgtcccgtcaagtcagcgtaatgctctgccagtgttacaaccaattaaccaattctgatTAGAAAAACTCATCGAGCATCAAATGAAACTGCAATTTATTCATATCAGGATTATCAATACCATATTTTTGAAAAAGCCGTTTCTGTAATGAAGGAGAAAACTCACCGAGGCAGTTCCATAGGATGGCAAGATCCTGGTATCGGTCTGCGATTCCGACTCGTCCAACATCAATACAACCTATTAATTTCCCCTCGTCAAAAATAAGGTTATCAAGTGAGAAATCACCATGAGTGACGACTGAATCCGGTGAGAATGGCAAAAGCTTATGCATTTCTTTCCAGACTTGTTCAACAGGCCAGCCATTACGCTCGTCATCAAAATCACTCGCATCAACCAAACCGTTATTCATTCGTGATTGCGCCTGAGCGAGACGAAATACGCGATCGCTGTTAAAAGGACAATTACAAACAGGAATCGAATGCAACCGGCGCAGGAACACTGCCAGCGCATCAACAATATTTTCACCTGAATCAGGATATTCTTCTAATACCTGGAATGCTGTTTTCCCGGGGATCGCAGTGGTGAGTAACCATGCATCATCAGGAGTACGGATAAAATGCTTGATGGTCGGAAGAGGCATAAATTCCGTCAGCCAGTTTAGTCTGACCATCTCATCTGTAACATCATTGGCAACGCTACCTTTGCCATGTTTCAGAAACAACTCTGGCGCATCGGGCTTCCCATACAATCGATAGATTGTCGCACCTGATTGCCCGACATTATCGCGAGCCCATTTATACCCATATAAATCAGCATCCATGTTGGAATTTAATCGCGGCCTCGAGCAAGACGTTTCCCGTTGAATATGGCTCATaacaccccttgtattactgtttatgtaagcagacagttttattgttcatgatgatatatttttatcttgtgcaatgtaacatcagagattttgagacacaacgtggctttcccccccccccctgcagagcaatatttcctgatacccctagggtaaatcatgggaaatggcgatcgccggagtttctcctgtttgctggagggctgtctgcaacatcttggtgctgaccacggaatcggtggcgaggttaaagaggggattagccagaatacctgccagcgaggtagcaaccaaagtagcgacaatgcccacctgtaggggacgcatgccgggtaaattccatttgatggccgggtaatttttgattacttcggacatttcctggggctccttcaccaccatcattttcaccacccggatgtagtagtagatggaaactacactggtaaccagaccaagtaggactaggccatacaatcccgattgccaaccggcccagaagatgtaaattttgccgaaaaagcccgccagaggaggaatgccccccaaggataataaacaaatgctcaagcccaaggttaacaaggggtctt

**Semi-continuous system for stability tests**

After 3 days of selection of the *Synechococcus* SmR transformant, an aliquot of liquid culture was withdrawn from the bioreactor and plated on MAD_low P_ petri dishes supplemented with spectinomycin 10 µg/mL. The petri dishes were firstly incubated at 30°C, white light LED panel (~150 µmol photons/m^2^/s) until colonies appeared, then transferred at room temperature. Six colonies were picked and tempered in 5 mL of liquid MAD_low P_ medium (without spectinomycin). After an initial lag period, cultures were diluted to 30 mL and grown in Falcon^®^ tubes at 24 °C, 100 µmol photons/m^2^/s, atmospheric CO_2_, under continuous shaking in semi-continuous conditions with τ=4 d (manually replacing 7,5 mL/day or 15 mL every other day). Samples were collected weekly to perform PCRs to verify the maintenance of the construct of interest. The experiment lasted a total of two months in liquid culture.

**PCR-based genomic DNA analysis**

***Picosynechococcus* transformants**

Genomic DNA templates were prepared as previously described (Formighieri & Melis, 2014) with some modifications. Pelleted cells were resuspended in 20 μL aliquot of Milli-Q water and mixed with an equal volume of 100% ethanol. The sample was then briefly vortexed before the adding of 200 μL aliquot of a 10% (w/v) Chelex-100 Resin (BioRad, USA). The solution was mixed again and then heated at 98 °C for 10 min to lyse the cells. Following centrifugation at 16,000 g for 10 min to pellet the cell debris, 1 μL of the supernatant was used as a genomic DNA template in a 10 μL PCR reaction mixture. Phusion DNA polymerase by Thermo Fisher (Waltham, USA) was used for genomic DNA PCR analyses, following manufacturer instructions. Primers used in the PCR analysis are listed in **Supplementary** **Table S1**.

***Synechocystis* transformants**

DNA was directly extracted from 1 uL of culture sample, adding 200 uL of chelex 5% and incubating at 95°C for 20 minutes. Samples were then put in ice for 20 minutes and centrifuged for 5 minutes at 13.000 rpm. 1 uL of supernatant was withdrawn and added to a 30 μL PCR reaction mixture. GoTaq^®^ Flexi DNA polymerase by Promega (Madison, USA) was used for genomic DNA analysis of *Synechocystis* samples, following manufacturer instructions. Primers used in the PCR analysis are listed in **Supplementary** **Table S1**.

| **Oligos name** | **Microorganism** | **DNA sequence 5’🡪3’** |
| --- | --- | --- |
| mrr5'_ext_fw | *Picosynechococcus sp. PCC 11901* | TGAATGATCAAATCGTCGAAG |
| mrr3'_ext_rv | *Picosynechococcus sp. PCC 11901* | CAATTATCAGAAGTGTCGGAG |
| mrr_int_genome_rv | *Picosynechococcus sp. PCC 11901* | TGAAGCTGTTGATGCCAAG |
| smr_int_rv | *Picosynechococcus sp. PCC 11901* | TCAGCAAGATAGCCAGATCA |
| kmr_int_fw | *Picosynechococcus sp. PCC 11901* | TCGTCACTCATGGTGATTTC |
| ns5’_fw | *Synechocystis sp. PCC 6803* | GCATTAGCCCGCCCTTTGAC |
| ns3’_rv | *Synechocystis sp. PCC 6803* | CCTCCTCTGGCGGGCTTTTT |

**Table S1:** Name and sequence of the oligonucleotides used as primers in this study.

**Absorption spectra analysis**

Cyanobacterial cultures were centrifuged at 10000 x g for 5 minutes, and pigments were extracted from cell pellet using DMSO. An incubation time of at least 90 minutes in a rotating mixer facilitated complete pigment extraction. Extracts were then diluted in acetone 95%, with the latter previously buffered with Na_2_CO_3_, to a final acetone concentration of 80%. The absorption spectra of the pigment extracts were measured with the Jasco V-730 spectrophotometer in the light visible range (350-750 nm).

**Thin-layer chromatography analysis**

Pigments were extracted in isopropanol from a cell pellet of transformed cells collected from the CSTR, and then they were separated by thin-layer chromatography (TLC) on Silica Gel 60 F254 Coated Aluminum-Backed TLC Sheets (VWR, USA) using an appropriate mobile phase (60% hexane, 20% chloroform, 20% acetone).

**SUPPLEMENTARY RESULTS**

**Transformation of *Picosynechococcus***

After incubating the transformation culture overnight, an aliquot was plated onto MAD_low P_ petri dishes with 10 µg/mL spectinomycin (SmR transformants) or 100 µg/mL kanamycin (bKT transformants) as confirmation of successful transformation. Antibiotic-resistant colonies began to emerge after approximately one week of incubation (**Figure S2**).


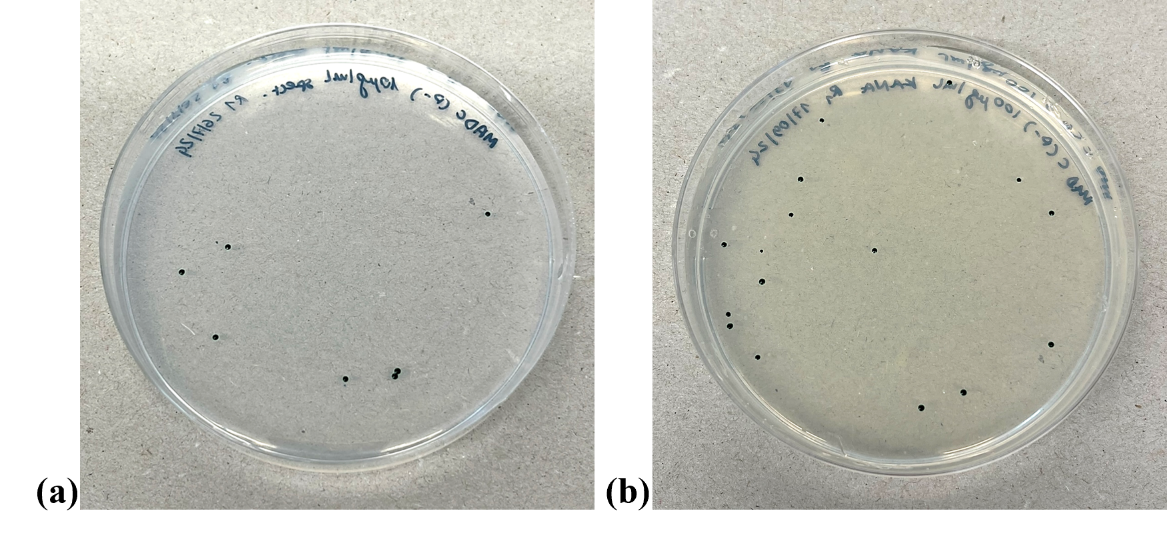


**Figure S2**: Colonies obtained after overnight transformation with **(a)** SmR construct, plated on 10 µg/mL spectinomycin MAD_low P_ medium and **(b)** bKT construct, plated on 100 µg/mL kanamycin MAD_low P_ medium.

Transformation with bKT construct resulted in a distinct pigment composition in the transformed cells compared to the wild type. Spectra analyses (Figure S3a) and TLC profiling (Figure S3b) (described in Supplementary Materials and Methods) enabled rapid monitoring and identification of transformant populations expressing the *bkt* gene and, therefore, synthetizing canthaxanthin.


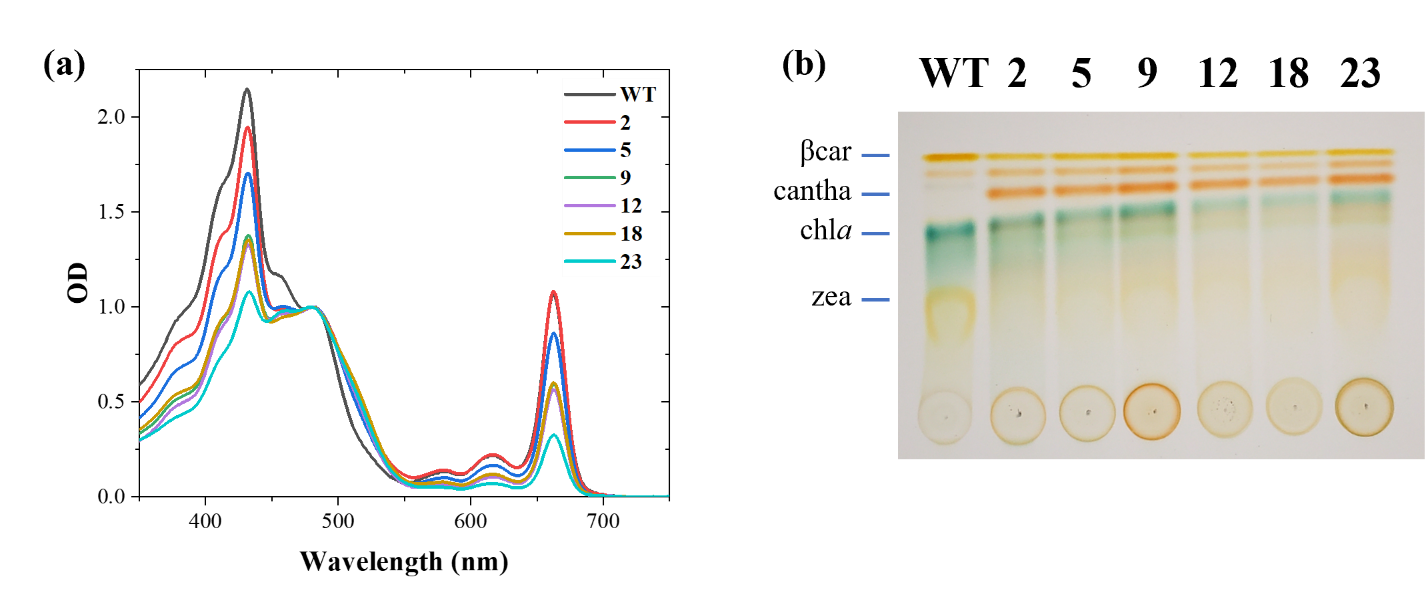


**Figure S3**: Analysis of the pigments accumulated in the transformed cells. **(a)** Absorption spectra of pigment extracts in the visible light range (350-750 nm) from WT and transformed cells collected at different time points (numbers represent the days of selection continuous cultivation). Spectra were normalized to maximum of absorption attributable to carotenoid in transformed cells (480 nm). **(b)** TLC analysis of isopropanol extracts of samples showed in **(a)**.

**Transformant stability in a semi-continuous system**

The homoplasmic colonies SmR1, SmR4 and SmR6, plated on selective solid medium after three days of selection in the CSTR, were isolated and grown in a semi-continuous system in the absence of antibiotic for 42 days. Growth was monitored via OD_750_ (Figure S4).


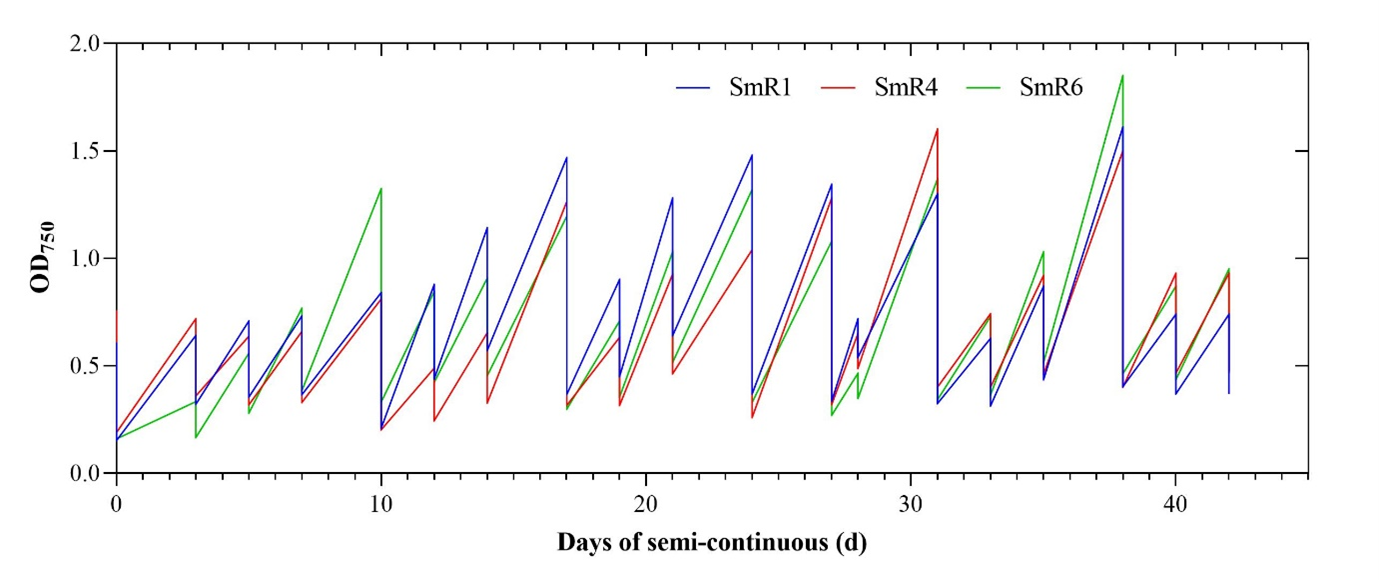


**Figure S4**: Monitoring of SmR1, SmR4 and SmR6 as OD_750_ over the 6-weeks period of semi-continuous growth.

**Validation of selection protocol on the model species *Synechocystis***

The selection protocol proposed in the present study was validated using the model species *Synechocystis*. PCR analyses, as depicted in **Figure S5,** confirm successful transformation and the attainment of a homoplasmic population of cells after 21 days of selection.


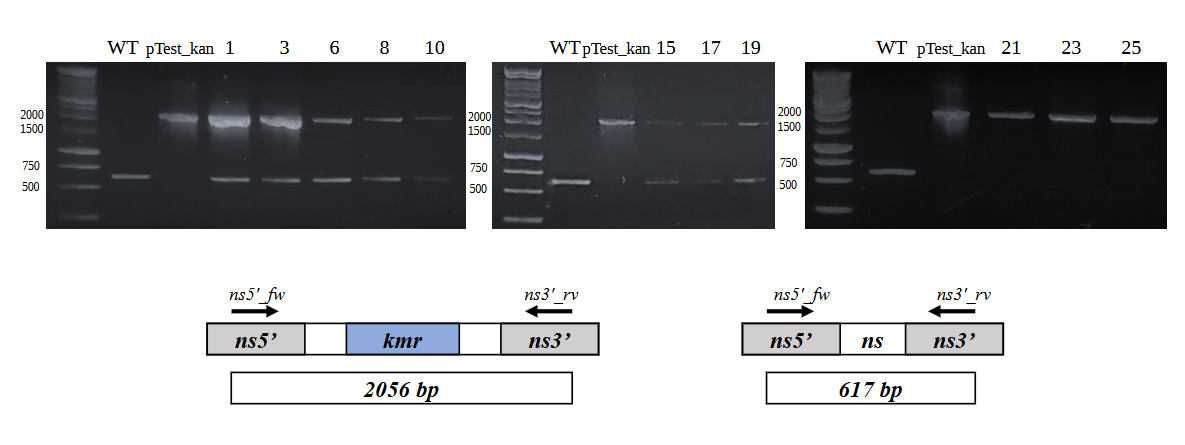
**Figure S5**: Genomic DNA PCR analyses testing for the presence of the transgenic or wild-type DNA sequences upon 1-25 days of selection in a continuous system of KmR *Synechocystis* transformants. Each PCR analysis comprised 30 cycles of DNA amplification, and the expected size of the PCR products was 2056 bp and 617 bp for the transgenic and WT sequences, respectively (primers ns5’_fw and ns3’_rv). Plasmid pTest_kan was used as positive control for the presence of *kmr*.
